# Editorial: Futuristic plant microbes biotechnology and bioengineering

**DOI:** 10.3389/fmicb.2024.1514583

**Published:** 2024-11-12

**Authors:** Surendra Sarsaiya, Ajar Nath Yadav, Pragya Tiwari, Ranjan Singh

**Affiliations:** ^1^Key Laboratory of Basic Pharmacology and Joint International Research Laboratory of Ethnomedicine of Ministry of Education, Zunyi Medical University, Zunyi, China; ^2^Department of Biotechnology, Dr. Khem Singh Gill Akal College of Agriculture, Eternal University, Rajgarh, Himachal Pradesh, India; ^3^Department of Horticulture and Life Science, Yeungnam University, Gyeongsan, Republic of Korea; ^4^Department of Microbiology, Dr. Rammanohar Lohia Avadh University, Ayodhya, India

**Keywords:** microbial species, plant-microbe interactions, sustainable agriculture, synthetic biology, sustainability

The field of plant-microbe interactions is rapidly evolving, and with advances in biotechnology and bioengineering, we are on the verge of unlocking new opportunities in agriculture, environmental sustainability, and health sciences. The integration of microbial biotechnology with plant systems has the potential to revolutionize crop productivity, nutrient efficiency, pathogen resistance, and climate resilience. As research continues, biotechnological interventions offer innovative solutions to global challenges, such as food security, ecosystem degradation, and sustainable energy production. This editorial explores the latest advancements in plant-microbe biotechnology, focusing on microbial applications in agriculture, bioengineering breakthroughs, and the future trajectory of this dynamic field.

Microbial communities are essential for plant health and development and interact with plant roots in the rhizosphere to promote nutrient uptake, enhance stress tolerance, and protect against pathogens. Beneficial plant-associated microbes, such as phosphate-solubilizing microorganisms (PSMs) and nitrogen-fixing bacteria, are increasingly being explored to reduce dependency on chemical fertilizers and promote sustainable agriculture (Jain et al.; Pang et al.). Phosphorus is a crucial element for plant growth, but it is often unavailable in soils due to its tendency to form insoluble compounds with calcium, iron, or aluminum. PSMs enhance phosphorus availability by secreting organic acids that solubilize these bound compounds, making phosphorus accessible to plants (Pang et al.). *Bacillus, Pseudomonas*, and *Aspergillus* species can significantly increase phosphorus uptake and improve plant growth and yield (Jain et al.). Symbiotic bacteria, such as *Rhizobium, Bradyrhizobium*, and *Azorhizobium*, play a vital role in nitrogen fixation by converting atmospheric nitrogen into ammonia, which plants can use. This natural process reduces the need for synthetic nitrogen fertilizers, thereby promoting agricultural and environmental sustainability (Pang et al.). Integrating such microbial functions into agricultural systems can increase crop yield, reduce chemical inputs, and develop resilient farming systems.

Recent advances in omics technologies, such as genomics, transcriptomics, proteomics, and metabolomics, have provided deeper insights into plant-microbe interactions (Jain et al.). These tools allow researchers to explore the complex molecular networks involved in plant-microbe symbiosis, pathogen defense, and nutrient acquisition. High-throughput sequencing technologies have enabled the detailed mapping of microbial communities in the rhizosphere, revealing the genetic underpinnings of symbiotic relationships. By analyzing microbial genomes, researchers can identify the key genes responsible for processes such as nitrogen fixation, phosphorus solubilization, and pathogen resistance (Jain et al.; Pang et al.). Proteomic analyses revealed the proteins involved in plant-microbe interactions, whereas metabolomic profiling uncovered the small molecules exchanged between plants and microbes. These tools provide a comprehensive understanding of the biochemical pathways that underpin these interactions and facilitate the development of microbial inoculants tailored to specific crop requirements (Jain et al.; Xie et al.). These technologies not only enhance our understanding of plant-microbe interactions but also pave the way for the development of precision agriculture solutions that optimize microbial functions to improve crop productivity.

Biotechnology is revolutionizing plant-microbe interactions by enabling the engineering of both plants and their associated microbes. Using synthetic biology, scientists are designing microbial strains with enhanced capabilities for nutrient solubilization, biocontrol, and stress tolerance (Jain et al.; Xie et al.). Microbial inoculants engineered to express specific traits such as drought tolerance or enhanced phosphorus solubilization can be applied to crops to improve their resilience under challenging environmental conditions. For example, *Streptomyces* spp. have been studied for their ability to produce antifungal compounds that protect crops from pathogens (Yang et al.). Advances in CRISPR-Cas technology has enabled precise editing of plant genomes to enhance their interactions with beneficial microbes. By modifying plant root exudates, researchers can selectively recruit beneficial microbes, fostering a more favorable rhizosphere environment (Xie et al.). These bioengineering strategies offer promising solutions for increasing crop yields, reducing the dependency on chemical inputs, and promoting sustainable agricultural practices.

As the global population continues to grow, there is an increasing need for sustainable agricultural practices that can meet rising food demands while minimizing the environmental impact. Plant-microbe biotechnology offers a range of solutions for promoting sustainable agriculture and enhancing climate resilience. Certain microbes play a role in sequestering carbon in the soil, thereby reducing greenhouse gas emissions and mitigating the effects of climate change. Enhancing microbial activity in agricultural soils can promote carbon storage and improve soil health (Jain et al.). Bioengineered microbes and microbial inoculants can enhance plant resilience to abiotic stresses such as drought and salinity. For instance, microbes that produce osmoprotectants or modify plant hormone pathways can help crops to withstand extreme weather conditions (Pang et al.; Yang et al.). These advancements not only increase agricultural productivity but also contribute to the development of climate-resilient farming systems.

**Figure 1:**
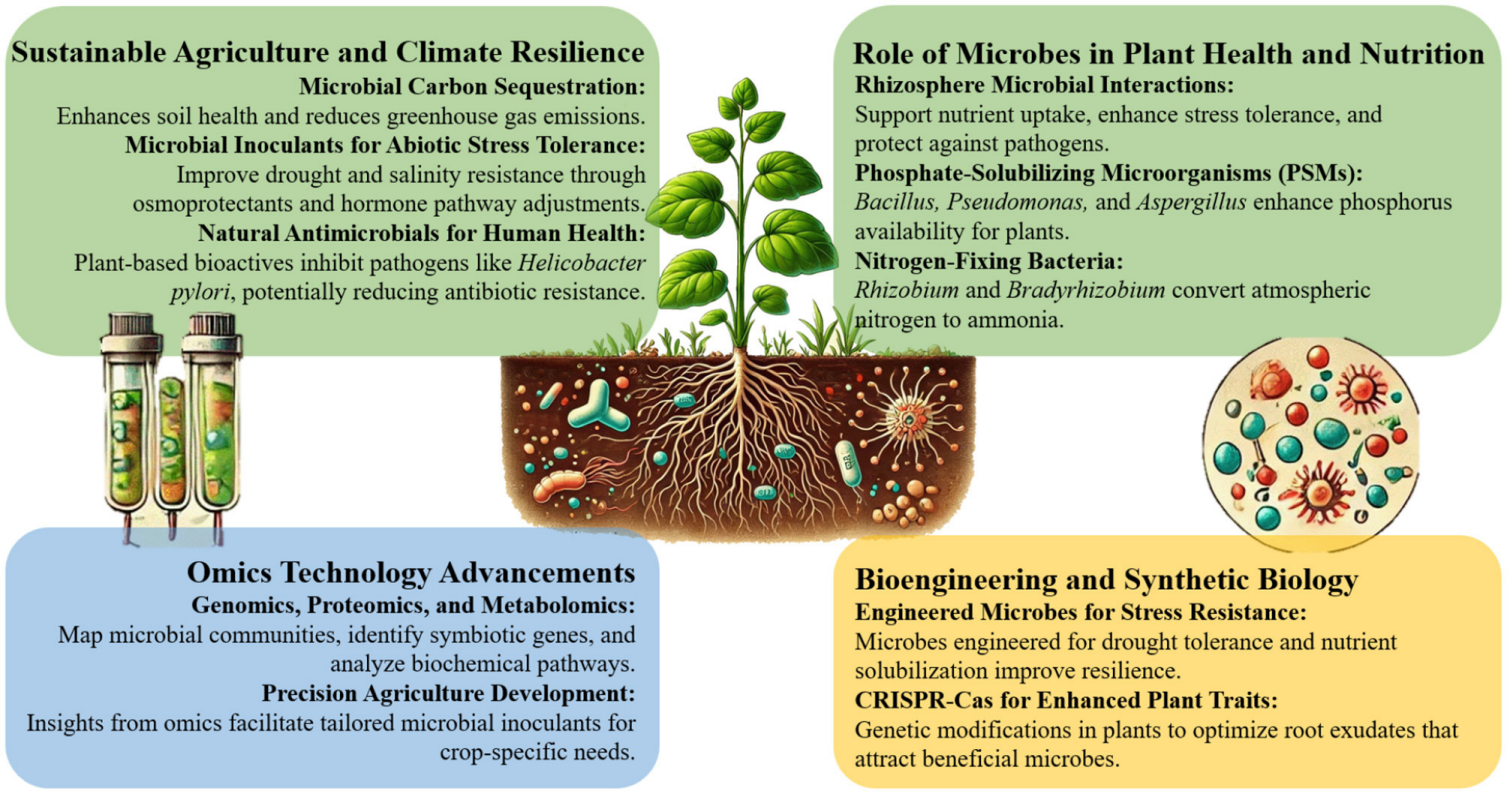
Plant-microbe interactions for sustainable agriculture using biotechnology and bioengineering.

The exploration of natural food resources and dietary ingredients for ameliorating *Helicobacter pylori* infection highlights the emerging potential of plant-microbe biotechnology in addressing human health challenges. Researchers are developing innovative alternatives to conventional antibiotic treatments by leveraging bioactive compounds found in vegetables, fruits, spices, and herbs. These natural resources have shown promise in inhibiting *H. pylori* colonization, reducing gastric inflammation, and enhancing the efficacy of antibiotics while minimizing the risks of bacterial resistance and gut microbiota imbalances. As the scientific understanding of plant-microbe interactions deepens, the application of these findings in biotechnology could revolutionize the development of sustainable, low-cost therapeutic strategies. This approach not only offers a promising avenue for treating persistent pathogens, such as *H. pylori*, but also underscores the broader potential of bioengineering microbial and plant systems for health and environmental resilience (Figure 1) (Wang et al.).

The future of plant-microbe biotechnology lies in the continued integration of omics technologies, synthetic biology, and precision agriculture. However, as we push the boundaries of microbial bioengineering, it is important to consider the ethical and ecological implications of releasing genetically modified organisms into the environment (Pang et al.). Although bioengineered microbes offer immense potential for improving crop productivity, their release into natural ecosystems raises concerns regarding unintended ecological consequences. Careful monitoring and regulatory oversight are essential to ensure the safe deployment of these technologies (Pang et al.). Public acceptance of genetically modified crops and microbial inoculants remains a challenge. Transparent communication about the benefits and risks of these technologies, along with the development of robust regulatory frameworks, is crucial for their widespread adoption (Jain et al.; Pang et al.).

In conclusion, the Research Topic was to publish high-quality research papers and review articles that focused on new insights, novel developments, current challenges, recent discoveries, recent advances, and future perspectives in the field. This Research Topic contains two research papers and three review papers that cover different aspects of plant-microbe interactions for plant health, growth, and productivity. Plant-microbe biotechnology has revolutionized agriculture by enhancing crop productivity, promoting environmental sustainability, and increasing resilience to climate challenges. Microbial communities, including phosphate-solubilizing organisms and nitrogen-fixing bacteria, play a vital role in improving nutrient uptake, stress resilience, and pathogen defense, thereby offering eco-friendly alternatives to traditional chemical inputs. Advances in omics technologies, such as genomics and metabolomics, have expanded our knowledge of these interactions, enabling the development of precision agriculture tailored to specific crop needs. Bioengineering and synthetic biology have further accelerated this progress, with CRISPR-Cas technologies allowing targeted editing of plant genomes to foster beneficial microbial relationships. Engineered microbial inoculants designed to enhance stress tolerance or nutrient solubilization present innovative solutions to challenges, such as drought and soil degradation. However, the potential ecological risks of bioengineered microbes and the complexity of scaling these solutions across diverse environmental conditions must be addressed. Ensuring that biotechnology is responsibly integrated into agricultural systems is essential to achieving long-term food security and fostering global sustainability.

